# Bis(μ-3,5-dimethyl-1,2,4-triazol-4-amine-κ^2^ *N* ^1^:*N* ^2^)bis­[dichlorido­cobalt(II)]

**DOI:** 10.1107/S1600536809021916

**Published:** 2009-06-17

**Authors:** Yun Gong, Jinghua Li, Yuchao Zhou, Jianbo Qin, Xiaoxia Wu

**Affiliations:** a Department of Chemistry, College of Chemistry and Chemical Engineering, Chongqing University, 400044 Chongqing, People’s Republic of China; b Department of Pharmaceutical Chemistry, College of Chemistry and Chemical Engineering, Chongqing University, 400044 Chongqing, People’s Republic of China

## Abstract

In the centrosymmetric dinuclear compound, [Co_2_Cl_4_(C_4_H_8_N_4_)_2_], the Co^II^ atom is coordinated by N atoms from two 3,5-dimethyl-1,2,4-triazol-4-amine ligands and two Cl atoms in a distorted tetra­hedral geometry. A six-membered ring is formed by four N atoms from two ligands and the two Co^II^ centers; the Co⋯Co distance is 3.756 (9) Å.

## Related literature

For related compounds, see: Cheng *et al.* (2007 ▶); Lavrenova *et al.* (1992 ▶); Liu *et al.* (2003 ▶); Nockemann & Meyer (2007 ▶).
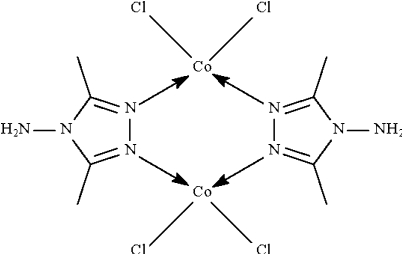


## Experimental

### 

#### Crystal data

[Co_2_Cl_4_(C_4_H_8_N_4_)_2_]
*M* *_r_* = 483.95
Monoclinic, 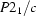

*a* = 6.7412 (10) Å
*b* = 12.2094 (16) Å
*c* = 11.4423 (14) Å
β = 97.8270 (10)°
*V* = 933.0 (2) Å^3^
*Z* = 2
Mo *K*α radiation
μ = 2.36 mm^−1^
*T* = 298 K
0.34 × 0.33 × 0.17 mm

#### Data collection

Siemens SMART CCD area-detector diffractometer
Absorption correction: multi-scan (*SADABS*; Sheldrick, 1996 ▶) *T*_min_ = 0.46, *T*_max_ = 0.67
4733 measured reflections
1638 independent reflections
1304 reflections with *I* > 2σ(*I*)
*R*_int_ = 0.023

#### Refinement

*R*[*F* ^2^ > 2σ(*F* ^2^)] = 0.032
*wR*(*F* ^2^) = 0.085
*S* = 1.07
1638 reflections
102 parameters
H-atom parameters constrained
Δρ_max_ = 0.43 e Å^−3^
Δρ_min_ = −0.58 e Å^−3^

### 

Data collection: *SMART* (Siemens, 1996 ▶); cell refinement: *SAINT* (Siemens, 1996 ▶); data reduction: *SAINT*; program(s) used to solve structure: *SHELXS97* (Sheldrick, 2008 ▶); program(s) used to refine structure: *SHELXL97* (Sheldrick, 20008); molecular graphics: *SHELXTL* (Sheldrick, 2008 ▶); software used to prepare material for publication: *SHELXTL*.

## Supplementary Material

Crystal structure: contains datablocks global, I. DOI: 10.1107/S1600536809021916/ng2584sup1.cif

Structure factors: contains datablocks I. DOI: 10.1107/S1600536809021916/ng2584Isup2.hkl

Additional supplementary materials:  crystallographic information; 3D view; checkCIF report

## Figures and Tables

**Table:** 

Co1—N2^i^	2.023 (3)
Co1—N1	2.030 (3)
Co1—Cl2	2.2154 (11)
Co1—Cl1	2.2382 (11)

**Table:** 

N2^i^—Co1—N1	107.55 (11)
N2^i^—Co1—Cl2	108.46 (9)
N1—Co1—Cl2	108.50 (9)
N2^i^—Co1—Cl1	109.60 (9)
N1—Co1—Cl1	109.49 (9)
Cl2—Co1—Cl1	113.10 (5)

Symmetry code: (i) 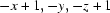.

**Fig. 1.**
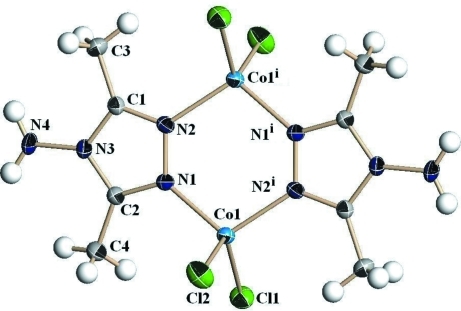
The structure of (I), with the atomic numbering scheme and displacement ellipsoids at the 30% probability level. [Symmetry codes: (i) -x + 1,-y,-z + 1.]

**Fig. 2.**
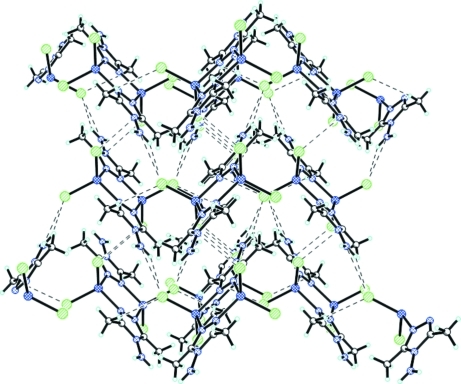
Three dimensional supramolecular architecture constructed by intermolecular hydrogen bonds. The dotted lines indicate the hydrogen bonds.

## supplementary crystallographic information

### Comment

The rational design and synthesis of novel coordination polymers is of current interest in the field of supramolecular chemistry and crystal engineering, not only because of their intriguing structural motifs but also because of their potential applications in catalysis, molecular adsorption, magnetism, nonlinear optics, and molecular sensing. 1,2,4-Triazole and its derivatives possess good coordination ability due to the hetercyclic nitrogen atoms in the structure. Many polymers of 3,5-dimethyl-1,2,4-triazol-4-amine (Dmatrz) have been synthesized. In 1992, Lavrenova reported a series of metal-Dmatrz complexes, such as CuCl_2_(Dmatrz)(0.5H_2_O), CdCl_2_(Dmatrz), Co(NO_3_)_2_(Dmatrz)_2_(H_2_O), Cu(NO_3_)_2_(Dmatrz)(0.5H_2_O), Ni(NO_3_)_2_(Dmatrz)_2_(H_2_O), Zn(NO_3_)_2_(Dmatrz)_2_, Cd(NO_3_)_2_(Dmatrz)_3_ (Lavrenova *et al.*, 1992). Other metal- Dmatrz complexes such as Cu(Dmatrz)SCN, Zn_2_(Dmatrz)_2_Cl_4_, Ag_3_(Dmatrz)_2_(NO_3_)_3_ have also reported (Liu, *et al.*, 2003; Cheng, *et al.*, 2007; Nockemann, *et al.*, 2007). However, so far coordination polymer constructed from CoCl_2_ and Dmatrz has never been reported. In the present word, we solvothermally synthesized a CoCl_2_-Dmatrz complex and it is reported here.

The molecular structure of the complex (I) (Fig. 1) has one one Co(II), one Dmatrz and two chlorine anions in its asymmetric unit. The Co(II) center is four-coordinated by four nitrogen atoms from two Dmatrz ligands and two chlorine atoms in a tetrahedral geometry. Each Dmatrz ligand links two Co(II) centers *via* its two neighboring nitrogen atoms with a Co···Co separation of 3.756 (9) Å (Fig.1). A six membered ring is formed *via* four nitrogen atoms from two Dmatrz ligands and two cobalt centers. The chlorine atoms can form hydrogen bonds with nitrogen atom from the uncoordinated amino group of Dmatrz. For example, The H4B···Cl2(ii) and N4···Cl2(ii) distances are 2.514 and 3.277 Å, respectively [Symmetry codes: (ii) -*x* + 1,-*y*,-*z* + 1]. The N4—H4B··· Cl2(ii) angle is 148.39 °.

### Experimental

A mixture of Dmatrz(0.05 mmol, 0.006 g), CoCl_2_(0.1 mmol, 0.024 g) and ethanol(5 mm l) was put into a Teflon-lined autoclave. The reaction mixture was heated at 120 centigrade for one and a half day, followed by slow cooling to room temperatrue and blue single crystals were collected. Elemental analyse found: C, 19.80; H, 3.39; N, 23.04; Cl, 29.28; Co, 24.45%.

### Refinement

H atoms were positioned geometrically and refined as riding atoms, with C—H = 0.96Å and *U*_iso_(H) = 1.5*U*_eq_(C) for methyl H atoms, N—H = 0.86Å and *U*_iso_(H) = 1.2*U*_eq_(C) for amino H atoms.

### Crystal data

**Table:**

[Co_2_Cl_4_(C_4_H_8_N_4_)_2_]	*F*(000) = 484
*M**_r_* = 483.95	*D*_x_ = 1.723 Mg m^−^^3^
Monoclinic, *P*2_1_/*c*	Mo *K*α radiation, λ = 0.71073 Å
Hall symbol: -P 2ybc	Cell parameters from 4733 reflections
*a* = 6.7412 (10) Å	θ = 2.5–25.0°
*b* = 12.2094 (16) Å	µ = 2.36 mm^−^^1^
*c* = 11.4423 (14) Å	*T* = 298 K
β = 97.827 (1)°	Block, blue
*V* = 933.0 (2) Å^3^	0.34 × 0.33 × 0.17 mm
*Z* = 2	

### Data collection

**Table:**

Siemens CCD area-detector diffractometer	1638 independent reflections
Radiation source: fine-focus sealed tube	1304 reflections with *I* > 2σ(*I*)
graphite	*R*_int_ = 0.023
φ and ω scans	θ_max_ = 25.0°, θ_min_ = 2.5°
Absorption correction: multi-scan (*SADABS*; Sheldrick, 1996)	*h* = −7→7
*T*_min_ = 0.46, *T*_max_ = 0.67	*k* = −14→14
4733 measured reflections	*l* = −13→8

### Refinement

**Table:**

Refinement on *F*^2^	Primary atom site location: structure-invariant direct methods
Least-squares matrix: full	Secondary atom site location: difference Fourier map
*R*[*F*^2^ > 2σ(*F*^2^)] = 0.032	Hydrogen site location: inferred from neighbouring sites
*wR*(*F*^2^) = 0.085	H-atom parameters constrained
*S* = 1.07	*w* = 1/[σ^2^(*F*_o_^2^) + (0.0358*P*)^2^ + 1.1376*P*] where *P* = (*F*_o_^2^ + 2*F*_c_^2^)/3
1638 reflections	(Δ/σ)_max_ = 0.001
102 parameters	Δρ_max_ = 0.43 e Å^−^^3^
0 restraints	Δρ_min_ = −0.58 e Å^−^^3^

### Special details

**Table:**

Geometry. All e.s.d.'s (except the e.s.d. in the dihedral angle between two l.s. planes) are estimated using the full covariance matrix. The cell e.s.d.'s are taken into account individually in the estimation of e.s.d.'s in distances, angles and torsion angles; correlations between e.s.d.'s in cell parameters are only used when they are defined by crystal symmetry. An approximate (isotropic) treatment of cell e.s.d.'s is used for estimating e.s.d.'s involving l.s. planes.
Refinement. Refinement of *F*^2^ against ALL reflections. The weighted *R*-factor *wR* and goodness of fit *S* are based on *F*^2^, conventional *R*-factors *R* are based on *F*, with *F* set to zero for negative *F*^2^. The threshold expression of *F*^2^ > σ(*F*^2^) is used only for calculating *R*-factors(gt) *etc*. and is not relevant to the choice of reflections for refinement. *R*-factors based on *F*^2^ are statistically about twice as large as those based on *F*, and *R*- factors based on ALL data will be even larger.

### Fractional atomic coordinates and isotropic or equivalent isotropic displacement parameters (Å^2^)

**Table:**

	*x*	*y*	*z*	*U*_iso_*/*U*_eq_	
Co1	0.59569 (7)	0.10736 (4)	0.40423 (4)	0.03186 (17)	
Cl1	0.84618 (15)	0.10010 (9)	0.29461 (10)	0.0545 (3)	
Cl2	0.44352 (17)	0.26855 (9)	0.39618 (12)	0.0652 (3)	
N1	0.7034 (4)	0.0747 (2)	0.5750 (2)	0.0360 (7)	
N2	0.6098 (4)	0.0089 (2)	0.6507 (2)	0.0345 (7)	
N3	0.8558 (4)	0.0937 (2)	0.7526 (2)	0.0341 (7)	
N4	0.9948 (5)	0.1233 (3)	0.8494 (3)	0.0505 (9)	
H4A	0.9879	0.0948	0.9174	0.061*	
H4B	1.0872	0.1700	0.8409	0.061*	
C1	0.7059 (5)	0.0212 (3)	0.7575 (3)	0.0328 (8)	
C2	0.8540 (5)	0.1240 (3)	0.6382 (3)	0.0365 (8)	
C3	0.6643 (6)	−0.0339 (3)	0.8663 (3)	0.0476 (10)	
H3A	0.6222	0.0194	0.9195	0.071*	
H3B	0.7834	−0.0698	0.9030	0.071*	
H3C	0.5602	−0.0871	0.8471	0.071*	
C4	1.0012 (7)	0.1982 (4)	0.5962 (4)	0.0576 (12)	
H4C	1.0375	0.1709	0.5233	0.086*	
H4D	1.1183	0.2022	0.6541	0.086*	
H4E	0.9437	0.2699	0.5837	0.086*	

### Atomic displacement parameters (Å^2^)

**Table:**

	*U*^11^	*U*^22^	*U*^33^	*U*^12^	*U*^13^	*U*^23^
Co1	0.0308 (3)	0.0312 (3)	0.0334 (3)	−0.00153 (19)	0.00350 (19)	0.0033 (2)
Cl1	0.0476 (6)	0.0600 (6)	0.0604 (6)	−0.0015 (5)	0.0239 (5)	−0.0066 (5)
Cl2	0.0540 (7)	0.0417 (6)	0.0976 (9)	0.0137 (5)	0.0018 (6)	0.0003 (6)
N1	0.0369 (17)	0.0385 (16)	0.0317 (16)	−0.0077 (13)	0.0015 (13)	0.0046 (13)
N2	0.0335 (16)	0.0330 (15)	0.0364 (16)	−0.0055 (12)	0.0026 (13)	0.0025 (13)
N3	0.0354 (16)	0.0355 (16)	0.0301 (15)	−0.0018 (13)	−0.0004 (12)	−0.0044 (12)
N4	0.051 (2)	0.067 (2)	0.0298 (16)	−0.0194 (17)	−0.0085 (14)	−0.0041 (15)
C1	0.0337 (19)	0.0314 (18)	0.0329 (19)	0.0008 (15)	0.0026 (14)	−0.0022 (14)
C2	0.0371 (19)	0.0365 (19)	0.0353 (19)	−0.0045 (15)	0.0030 (15)	0.0000 (15)
C3	0.058 (3)	0.049 (2)	0.036 (2)	−0.0039 (19)	0.0052 (18)	0.0071 (17)
C4	0.061 (3)	0.067 (3)	0.045 (2)	−0.029 (2)	0.007 (2)	0.000 (2)

### Geometric parameters (Å, °)

**Table:**

Co1—N2^i^	2.023 (3)	N4—H4A	0.8600
Co1—N1	2.030 (3)	N4—H4B	0.8600
Co1—Cl2	2.2154 (11)	C1—C3	1.475 (5)
Co1—Cl1	2.2382 (11)	C2—C4	1.472 (5)
N1—C2	1.310 (4)	C3—H3A	0.9600
N1—N2	1.394 (4)	C3—H3B	0.9600
N2—C1	1.312 (4)	C3—H3C	0.9600
N2—Co1^i^	2.023 (3)	C4—H4C	0.9600
N3—C1	1.350 (4)	C4—H4D	0.9600
N3—C2	1.358 (4)	C4—H4E	0.9600
N3—N4	1.397 (4)		
			
N2^i^—Co1—N1	107.55 (11)	N2—C1—N3	108.3 (3)
N2^i^—Co1—Cl2	108.46 (9)	N2—C1—C3	127.4 (3)
N1—Co1—Cl2	108.50 (9)	N3—C1—C3	124.3 (3)
N2^i^—Co1—Cl1	109.60 (9)	N1—C2—N3	108.1 (3)
N1—Co1—Cl1	109.49 (9)	N1—C2—C4	127.5 (3)
Cl2—Co1—Cl1	113.10 (5)	N3—C2—C4	124.4 (3)
C2—N1—N2	107.7 (3)	C1—C3—H3A	109.5
C2—N1—Co1	126.2 (2)	C1—C3—H3B	109.5
N2—N1—Co1	125.3 (2)	H3A—C3—H3B	109.5
C1—N2—N1	107.7 (3)	C1—C3—H3C	109.5
C1—N2—Co1^i^	126.9 (2)	H3A—C3—H3C	109.5
N1—N2—Co1^i^	124.1 (2)	H3B—C3—H3C	109.5
C1—N3—C2	108.1 (3)	C2—C4—H4C	109.5
C1—N3—N4	124.1 (3)	C2—C4—H4D	109.5
C2—N3—N4	127.6 (3)	H4C—C4—H4D	109.5
N3—N4—H4A	120.0	C2—C4—H4E	109.5
N3—N4—H4B	120.0	H4C—C4—H4E	109.5
H4A—N4—H4B	120.0	H4D—C4—H4E	109.5

Symmetry codes: (i) −*x*+1, −*y*, −*z*+1.
